# Editorial: Genetic engineering in farm animals

**DOI:** 10.3389/fgene.2023.1155201

**Published:** 2023-02-21

**Authors:** Bjoern Petersen

**Affiliations:** Institute of Farm Animal Genetics, Friedrich-Loeffler-Institut, Neustadt am Ruebenberge, Germany

**Keywords:** genetic engineering, CRISPR-Cas, livestock, gene editing, MSTN, IFNAR, eggshell

Worldwide agriculture faces enormous challenges. Global warming, a growing world population with a constantly growing need for high-quality food, increasing demand for animal welfare, and reducing the ecological footprint represent the greatest challenges for the present and the near future.

Deciphering the function of the CRISPR-Cas9 system and adapting it for genome editing of the mammalian genome has revolutionized the genetic engineering of farm animals and offers novel opportunities to address these challenges (Jinek et al., 2012; Cong et al., 2013; Jinek et al., 2013). Besides their use for food production, livestock serves as an important animal model for human diseases and potential organ donors as they share high similarities with humans regarding physiology, anatomy, genetics, and size. This Frontiers in Genetics Research Topic includes six manuscripts that span from gene-edited pigs that avoid castration to prevent boar taint to basic research to identify potential targets for genetic engineering in chickens to increase the stability of eggshells. The castration of piglets with or without anesthesia is increasingly being criticized as it is associated with pain, stress, and an increased risk of infection. Disrupting porcine Kisspeptin-1 (KISS1) is hypothesized to delay or abolish puberty by inducing variable hypogonadotropism and thus preventing the need for castration. To test this hypothesis, Florez et al. generated the first KISS1-edited large animal using CRISPR/Cas9-ribonucleoproteins and single-stranded donor oligonucleotides. Though follow-on research will be necessary to evaluate the efficacy of this approach, this proof-of-principle study gives a good example of how genetic engineering could be employed to address urgent problems in livestock production. The knockout of Myostatin (MSTN), a negative regulator of muscle growth, is accompanied by a significant increase in skeletal muscle mass and has been discussed to increase the meat yield from livestock species (Crispo et al., 2015; Wang et al., 2015; Yu et al., 2016; Wang and Petersen, 2022). Several MSTN-KO livestock have been produced, but the visceral development remains unclear. Pei et al. provide the first evidence that MSTN-KO pigs show no significant difference in the size and average weight or length of visceral organs in adult pigs. These findings are important for the further application of this technology.

The importance of genetically engineered livestock is further displayed in the article by Davies et al. that generated a unique type I Interferon Receptor knockout sheep model for viral immunology and reproductive signaling. The mini-review by Greising et al. reviews the recent developments and options for the treatment of volumetric muscle loss in gene-edited pigs, while Ruan et al. provide a comprehensive opinion on the recent developments in altering the pig genome.

The establishment and culture of PGCs enabled the targeted genetic modification of the chicken genome to address productive traits such as eggshell strength and thickness which are critical factors in reducing egg breaking and preventing economic losses (Oishi et al., 2016; Atsuta et al., 2022). To identify potential targets for genetic engineering, Wu et al. employed transcriptional sequencing and proteomics to investigate the differences between the uteruses of laying hens with high- and low-breaking eggshells. They identified the KRT14 gene which may promote calcium metabolism and the deposition of calcium carbonate in eggshells.

Since its advent, CRISPR-Cas9 has proven its disruptive potential for animal breeding. A multiverse of different CRISPR variants has been developed in the last 10 years increasing the efficiency of precisely altering the genome of livestock species and reducing the number of unwanted mutations (Cong et al., 2013; Fu et al., 2014; Kleinstiver et al., 2015; Kleinstiver et al., 2016; Anzalone et al., 2019; Walton et al., 2020). Except for the European Union, most countries worldwide follow liberal regulations for novel breeding tools and consider approval of gene-edited livestock to integrate them into breeding programs to produce food for human consumption. A legal and ethical debate is still ongoing, but the assessment of genetically modified farm animals is moving more and more from a technology-based to a product-based risk assessment. With all the uncertainties and risks associated with the use of genetic engineering, we should also consider the risks of not using the new breeding technologies.
